# Reshaping the vector control strategy for malaria elimination in Ethiopia in the context of current evidence and new tools: opportunities and challenges

**DOI:** 10.1186/s12936-018-2607-8

**Published:** 2018-12-05

**Authors:** Taye Gari, Bernt Lindtjørn

**Affiliations:** 1 School of Public Health, College of Medicine and Health Sciences, Hawassa University, Hawassa, Ethiopia; 2 Centre for International Health, University of Bergen, Bergen, Norway

**Keywords:** Elimination, Ethiopia, Malaria, Vector control

## Abstract

The core vector control measures, long-lasting insecticidal nets (LLINs) and indoor residual spraying (IRS), reduce the risk of malaria infection by targeting indoor biting mosquitoes. These two interventions are found to be effective in malaria control, but not sufficient to eliminate malaria. The main challenges with LLINs and IRS are insecticide resistance, misuse of the interventions, host behaviour, such as staying out-door during early night or sleeping outdoor without using protective measures, and vector behaviour including feeding on bovine blood, outdoor biting and outdoor resting. Therefore, for complete interruption of malaria transmission in a defined area there is a need to consider a variety of interventions that can help prevent out-door as well as indoor malaria transmission. In Ethiopia, to achieve the malaria elimination goal, a mix of vector control tools, such as intensifying the use of LLINs and IRS, and supplemented by use of ivermectin administration, zooprophylaxis, odour-baited mosquito trapping, improving housing and larva control measures tailored to the local situation of malaria transmission, may be needed.

## Background

Malaria is a parasitic infectious disease, and transmitted by female *Anopheles* mosquitoes [1, 2]. The disease is a global public health problem, particularly in sub-Saharan Africa [3]. Cognizant of the serious health, economic and social challenges posed by the disease in malaria endemic countries, the Roll Back Malaria (RBM) Partnership was launched in 1998 with the goal of halving world’s malaria burden by 2010 [4]. The evaluation of RBM shows that the global malaria incidence has decreased by 37% between 2000 and 2015 [5]. However, the decline has stalled between 2015 and 2016, and malaria remains a global public health problem in 2016 [3]. The reduction in malaria has mainly been attributed to improved case management, and scale-up of long-lasting insecticidal nets (LLINs) and indoor residual spraying (IRS) [6]. In 2015, a re-organized global effort for malaria control and elimination has adopted a Global Technical Strategy for Malaria 2016–2030 (GTS) with targets to reduce malaria incidence and mortality by at least 90%, eliminate malaria from at least 35 countries and prevent malaria re-establishment from malaria free countries by 2030 [7]. In the GTS, vector control measures remains a core intervention strategy for the global malaria elimination programme [8].

Vector control refers to measures of any kind against malaria transmitting mosquitoes, intended to limit the ability or vectorial capacity to transmit the disease [9]. Vectorial capacity is defined as the number of new infections that the population of a given vector would induce per case per day at a given place and time, assuming that the human population is, and remains, fully susceptible to malaria [9]. The modified formula for vectorial capacity is:

$$ C = \frac{{ma^{2} P^{n} }}{{ - \,\log_{e} P}} $$

where, C; stands for vectorial capacity, ma; number of bites per human per night by available vectors, a; feeding frequency × human blood index, P; survival probability and n; incubation period of parasite in the mosquito.

This formula indicates that vector control measures that kill mosquitoes or shorten the survival of mosquitoes, such as IRS and LLINs, can play major roles in limiting the ability of mosquitoes to transmit malaria. This theoretical basis had been used to inform policy makers to select the most appropriate malaria vector control measures. For example, the use of IRS with dichloro-diphenyl-trichloroethane (DDT) in the 1950s and 1960s malaria eradication interventions was found to be effective [10].

The two main vector control interventions, LLINs and IRS, are used to target mosquitoes biting and resting indoor, but cannot prevent outdoor malaria transmission in the presence of efficient vectors that prefers to feed on human blood outdoors, or bite indoors and rest outdoors [11]. The challenges with the use of LLINs and IRS are insecticide resistance and residual transmission (remaining low malaria transmission in the presence of high coverage of LLINs and IRS to which the local vector is fully susceptible) [9, 12]. The remaining low malaria transmission is attributed to mosquito resting and biting behaviour (biting outdoors, biting indoors and leaving the house before exposure to a lethal dose of insecticides, and feeding on animals); and human behaviour, such as sleeping or staying outdoors at night [13]. Hence, the scale-up of LLINs and IRS can result in substantial decline in malaria burden during the control phase, but fail to completely interrupt malaria transmission [13, 14]. In such circumstance, there is a need to use a combination of vector control tools that target malaria vector at different life cycle stages in addition to the LLINs and IRS [14].

## Malaria control in Ethiopia

In Ethiopia, approximately 68% of the land mass of the country have favourable conditions for malaria transmission, and 60% of the population are at risk of malaria [15, 16]. The transmission of malaria is unstable and seasonal, usually characterized by frequent focal, and sometimes large scale epidemics [15, 17]. *Plasmodium falciparum* (70%) and *Plasmodium vivax* (30%) are the common causes of malaria [18]. The primary malaria vector in the country is *Anopheles arabiensis* and the secondary vector is *Anopheles pharoensis* [17]. Studies have shown that *An. arabiensis* and *An. pharoensis* are both endophagic and exophagic (indoor and outdoor feeding), endophilic and exophilic (indoor and outdoor resting), and have behavioural preferences for anthropophilia and zoophilia (human and bovine blood) [11, 19].

**Fig. 1:**
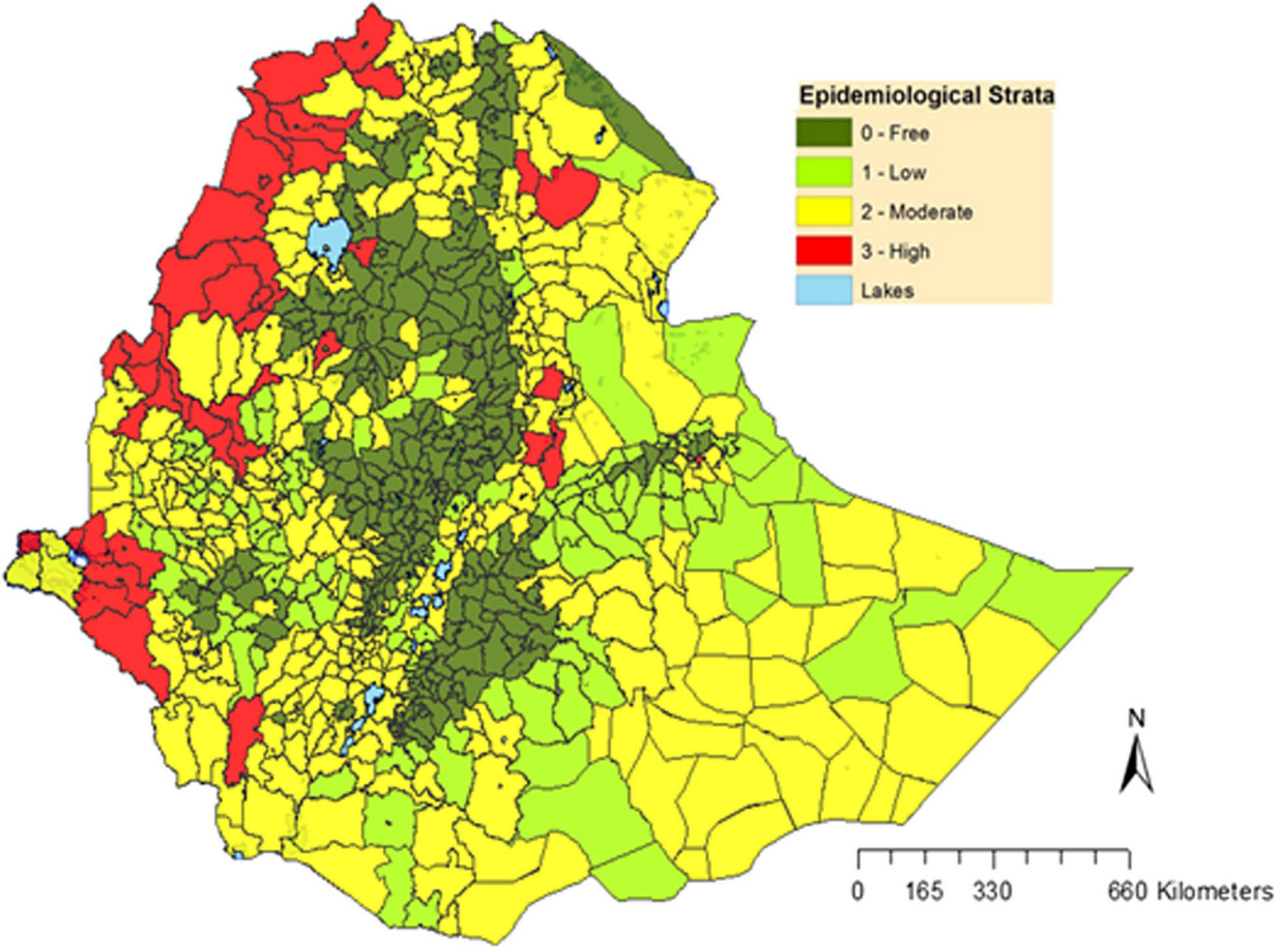
Map of malaria strata by districts in Ethiopia (©2017), adopted from National Malaria Elimination Road Map [18]

The national malaria control programme was initiated as the ‘Malaria Eradication Service’ in 1959 [20], and employed blanket IRS of houses with DDT and treatment of cases with chloroquine [20]. However, the country failed to attain its malaria eradication goals, and the approach was changed from malaria eradication to a malaria control programme in early 1980s [15]. This control programme was implemented as vertical programme, and case management and selective indoor residual spraying with DDT were the main malaria control measures [20]. In 1993, the vertical programme was reorganized through integration of malaria control in the health services in a decentralized approach during which scanty targeted IRS and case management were used as a tool to control malaria [15]. Since 2005, in line with RBM initiative, Ethiopia has scaled-up malaria control interventions which now include prompt diagnosis and treatment of cases with artemisinin-based combination therapy (ACT), epidemic prevention and control, information education communication and selective application of vector control tools, particularly the use of LLINs and IRS [21]. This coordinated effort has substantially reduced the malaria burden in the country between 2005 and 2015 [5]. Despite the low level of national confirmed malaria prevalence (0.5%), large variation exists among the regions of the country as shown in Fig. 1 [16].

Cognizant of the decline in malaria prevalence, the country has launched malaria elimination road map to eliminate malaria by 2030 [18]. In the meantime, the variation in malaria transmission risk between districts was taken into consideration, and the intended approach is to attempt sub-national elimination before embarking to national malaria elimination [18]. The proposed vector control interventions in the elimination road map (unpublished document) were intensifying LLINs and IRS, and selective application of larva control measures with source reduction and larviciding [22]. Modelling studies have suggested that in areas where *An. arabiensis* is the dominant vector, IRS or LLINs or their combinations, may only provide a limited protection [23]. Therefore, the question is if the currently used vector control interventions are sufficient to eliminate malaria in Ethiopia? Or does Ethiopia need to re-shape these interventions?

The aim of malaria elimination is reducing transmission to a levels less than self-sustaining called basic reproduction number (Ro), the number of secondary cases originating from single index case, to below one [24]. A review by Ferguson suggests that to reduce Ro < 1 requires an over 99% reduction in malaria transmission intensity to achieve malaria elimination [24]. It is also well documented that LLINs and IRS (main proposed vector control for malaria elimination in Ethiopia) are effective in malaria control, but not enough to eliminate malaria [25].

This paper evaluates the possible supplementary vector control options to re-shape the proposed interventions in the malaria elimination road map of Ethiopia, while LLINs and IRS should remain as main malaria elimination tools. A literature search was performed in PubMed databases, the grey literature, the World Health Organization (WHO) and the Ethiopian Ministry of Health archives in June 2018 using the key words “vector control” and “malaria elimination”. To describe the successes and challenges of vector control strategies for malaria control and elimination in the past, the literature search was done without time limit.

## Methods of vector control interventions

### Long-lasting insecticidal nets (LLINs)

Studies have shown that the use of LLIN is an effective tool in preventing malaria transmission, and has been implemented as one of the main vector control intervention in malaria endemic countries [26, 27]. High coverage and utilization of LLINs is recommended to achieve malaria elimination goals [22]. In Ethiopia, vector resistance to the insecticide chemicals on the LLINs, misuse and high bed net loss are the serious challenges of LLINs [22]. For example a cohort study from south-central Ethiopia showed that 60% of the bed nets were lost after 1 year of mass distribution [28]. On the other hand, the 2016 Ethiopia Malaria Indicator Survey has shown that only 62% of LLINs in the households were used the night before the survey [16].

Recent trials using LLINs with permethrin (a pyrethroid) and pyriproxyfen had increased efficacy compared with LLINs treated with permethrin alone [29]. Therefore, there is a need to use the new LLINs coated with a long-lasting chemical, such as piperonyl butoxide (PBO) combined with pyrethroid insecticide to overcome resistance [30]. PBO has no intrinsic pesticidal activity, but it inhibits certain metabolic enzymes within the mosquito that detoxify insecticides before they can have toxic effect on the mosquito. Thus, PBOs enhance pyrethroids on the LLINs to have toxic effect to mosquitoes (reversal resistance) [30]. In a recent study from the Rift Valley in Ethiopia, susceptibility to deltamethrin was restored after exposure of *An. arabiensis* to PBO implicating the role of mixed function oxidases in the resistance of this insecticide [31]. There is a need to test if the efficacy of this new LLINs in areas where the vector is *An. arabiensis*. Moreover, as LLINs remains the mainstay in the malaria elimination goal, due emphases should be given not only to universal coverage but also to correct use and replacement of the lost LLINs.

### Indoor residual spraying (IRS)

The feeding and resting behaviour of *Anopheline* mosquitoes affect the effectiveness of IRS and LLINs (target indoor biting and resting mosquitoes). A study conducted in south-central Ethiopia has shown greater outdoors than indoors mosquito human-biting activities during the early part of the night, even if human biting occurs throughout the night [11]. Indoor residual spraying is the application of long-acting chemical insecticides on the interior walls and roofs of houses to kill adult mosquito resting on these surfaces [32]. Studies have shown that IRS is effective in reducing new malaria infections and mortality from malaria [32–34], and has been implemented as one of the major vector control measures. The use of indoor residual spraying with DDT had played a central role in the success of the 1950s and 1960s WHO-led malaria eradication campaign, and remains the mainstay in the global coordinated effort to control and eliminate malaria [7, 20]. The WHO recommended insecticide for IRS against malaria vector are grouped under: Organochlorine (DDT), Organophosphates (malathion, fenitrothion, pirimiphos-methyl WP, pirimiphos-methyl CS), Carbamates (bendiocarb, propoxur) and Pyrethroids (alpha-cypermethrin WP, alpha-cypermethrin WG, bifenthrin, cyfluthrin, deltamethrin SC, deltamethrin WP, etofenprox and lambda-cyhalothrin) [35].

In Ethiopia, *An. arabiensis* is highly resistant to DDT, malathion and deltamethrin, but susceptible to pirimiphos methyl, bendiocarb and propoxur [22]. Moreover, the rapid emergence of insecticide resistance is a threat to the future malaria elimination plan [36]. Therefore, there is a need to implement insecticide management and have new chemical insecticide on track, as IRS remains a key intervention tool in the malaria elimination plan of Ethiopia.

### Improving housing

Poor housing conditions, such as the presence of open eaves or holes allowing mosquito entrance are risk factors for malaria infection [37, 38]. Meanwhile, mosquito-proofed housing can limit the entrance of mosquito into the house and reduce the risk of indoor malaria transmission that occurs before sleeping time [38]. Among others, screening and a general housing improvement had been used as supplementary component in the malaria elimination strategy in the developed world [25]. A randomized controlled trial in south Ethiopia has shown that almost 50% reduction in indoor vector density, entomological inoculation rate and malaria incidence among houses in which windows and doors screened with wire-mesh [39]. This strategy of house improvement can be implemented using locally available materials, is affordable, locally acceptable and also durable [39]. Thus, it can be one of the supplementary methods to LLINs or IRS to limit mosquito bite rate.

### Zooprophylaxis and insecticide-treated livestock

Zooprophylaxis is the use of animals to divert the blood-seeking mosquito from human hosts; whereas an insecticide-treated livestock refers to the treatment of animals with appropriate insecticides to control mosquitoes feeding on animals [19, 40]. The effectiveness of these interventions is determined by local vector behaviour, such as zoophilic and exophilic vectors, habitat separation between human and animal quarters, and augmenting zooprophylaxis with insecticide treatment of animals or co-intervention of LLINs and/or IRS [40, 41]. For example, a study from south Ethiopia showed the primary malaria vector, *An. arabiensis,* feeds both on human and bovine blood [19]. Hence, targeting mosquitoes feeding on cattle using zooprophylaxis or using an insecticide-treated livestock can be a supplementary vector control tools in an effort to eliminate malaria. A mathematical model combining data from Pakistan and Ethiopia has shown the intervention can decrease malaria transmission in areas where zoophilic vectors, such as *An. arabiensis,* are the main malaria vectors [40]. Moreover, a recent systematic review also showed that this intervention can be used in East Africa [42], and could be an option to reduce malaria transmission in Ethiopia.

### Ivermectin administration to humans

Ivermectin (IVM) is an effective medicine against a variety of parasites and vectors, and is used to treat lymphatic filariasis and onchocerciasis [6]. Studies have shown that IVM can kill *Anopheles* mosquitoes feeding on human blood, and simultaneously it can kill *Plasmodium* parasites [43, 44]. An experimental study on human volunteers showed higher mosquito mortality in the IVM group compared to the control [45]. Consistently, a recent study in Greater Mekong sub-region has shown that IVM mass drug administration has a potential to prevent malaria transmission [46]. Moreover, a study conducted in West African countries showed a single dose of IVM administration substantially reduced malaria transmission [47]. IVM drug is safe at high doses as 300 μg/kg/day for 3 days, and is an option to control exophagic or exophilic vectors [48]. It has been shown that with fewer MDA (mass drug administration) rounds, ivermectin can increase the impact of MDA with an ACT on malaria transmission, and thus adding IVM could sustain impact on malaria prevalence even if the MDA coverage is reduced [43]. Therefore, the WHO is considering the use of mass IVM administration to humans as complementary tool to control outdoors biting mosquitoes [43]. However, it has not yet been tried in Ethiopia, and there is a need for research to inform policy makers on the use of IVM in malaria elimination plan.

### Odour-baited mosquito trapping systems

A synthetic odour blend consisting of mosquito attractants can attract more mosquitoes than humans, and can be used as means of trapping and killing mosquitoes [49]. This system can attack both male and female mosquitoes, and result in reduction of malaria vector density [25]. Studies have shown that an odour-baited station can be used as a trap and the contamination kills mosquitoes escaping the trap shortly afterwards [50]. Odour-baited traps can supplement LLINs and can reduce malaria transmission. It is an affordable tool to help malaria elimination plan in Africa [51]. Moreover, a cluster randomized trial in Kenya showed low malaria prevalence in clusters using solar-powered odour-baited mosquito trapping system compared to control clusters [52], and can be a potential option to reduce outdoor biting. However, odour-baited mosquito trapping systems are currently more costly than other vector control measures [52]. Future studies should consider evaluating the efficacy and cost effectiveness of odour-baited mosquito trapping systems in Ethiopia.

### Space spraying

Male mosquitoes aggregate in swarms, where they compete for the attention of female mosquitoes visiting them in search of a mate. Swarms usually happen at sunset and at locations that can be mapped [53]. A review on space spraying targeting these aggregations with hand-held insecticide aerosol spray was found to be effective [54]. The WHO recommends space spraying in buses, trains and airplanes at their departure from malaria endemic countries as a strategy to prevent re-introduction of malaria into countries where malaria is eliminated [55], and to control epidemic in urban area or refugee camps [56]. Further investigation is needed to generate evidence to recommend space-spraying as a supplementary tool in an effort to eliminate malaria from Ethiopia.

### Insecticide-treated plastic sheeting (ITPS)

Plastic sheeting (polythene tarpaulins) impregnated with insecticide is usually used as a shelter when erected in refugee camps [57]. Moreover, permethrin-treated blankets, top sheets and clothing are also used as a personal protection in military camps [58]. Insecticide-treated plastic sheeting is effective in the areas of vector resistance to permethrin, as a result of the repellent effect of permethrin. A randomized controlled trial in a refugee setting in Sierra Leone showed that ITPS is safe, effective and a long-lasting measure to be used in emergency situation [57]. The effectiveness of ITPS is comparable to that of LLINs. Hence, it can be an option in emergencies.

### Mosquito repellents

Repellent lotions may provide personal protection against outdoor biting mosquitoes, in situations where LLINs or IRS cannot be used. A cluster randomized trial from Ethiopia showed that mosquito repellents are potentially effective to reduce malaria infection [59]. However, a cluster randomized controlled trial in the Greater Mekong Sub-region has shown that mass distribution of repellents did not contribute to reduction of malaria [60]. A recent Cochrane systematic review has shown that there is insufficient evidence concerning the effectiveness of mass distribution of repellents for malaria prevention [61]. Thus, better evidence from further studies with rigorous methods is needed in Ethiopia.

### Larval control measures

Larval control measure is the use of environmental manipulation to reduce vector breeding sites or use of chemical or biological larvicidal methods to eliminate the larval stage of mosquitoes [56]. This could be an important tool to reduce risk of vector biting in low malaria transmission settings, especially those planning to eliminate malaria [55]. The WHO recommends larval control measures as complementary to LLINs or IRS in areas where mosquito breeding sites are few, fixed and findable [56]. Larval control could use chemical larvicides, biological or environmental methods.

### Chemical larvicides

Temephos (Abate^®^) is the most common larvicidal chemical in use in Ethiopia [62]. It is effective and safe for use in water [63], and a potential complementary tool to control malaria in areas where breeding sites are findable places, such as urban areas, dams, irrigation canals and other developmental areas. The other chemical candidate larvicide is *Bacillus thuringiensis H*-*14*, a bacteria larvicidal based on insecticidal crystal proteins that are specifically toxic to mosquitoes [63]. It is effective and is a potential option in Ethiopia [22]. However, the chemical and spray equipment are expensive and, thus not widely used.

### Biological methods

Larvivorous (larva-eating) fish feed on mosquito larvae, and have been used around the world in attempts to control malaria [64]. The advantages of using larvivorous fish are that it is a cheaper and more environmentally friendly alternative to insecticide-based measures to control malaria [64]. The use of larvivorous fish in combination with IRS and case treatment in India was found to be effective in malaria control [65]. A systematic review has shown that the use of larva-eating fish in concrete tanks was effective in malaria control [54]. However, countries should not invest in fish stocking as a standalone or supplementary larval control measure in any malaria transmission areas outside the context of the research included in this review. The limitations are the rearing, transportation and stocking that require special care [64].

### Environmental methods

Environmental methods of vector control include filling, drainage of stagnant swampy areas, and removing or destroying mosquito breeding sites [64]. For example a randomized controlled trial in north Ethiopia on source reduction using filling and draining of breeding sites done by the community mobilization was effective in reducing the vector density [66]. The advantage is it kills larvae without polluting the environment, but the limitation is that it requires large human resource [22]. The effectiveness of the intervention in vector control depends on the active participation of the community, regular implementation and close supervision [22].

### Opportunities

Malaria elimination is more expensive than control programmes, as it requires advanced tools to diagnose all persons with malaria infection, including asymptomatic carriers, a strong surveillance system (entomological and epidemiological), a predictable strong domestic funding, a strong political commitment, political stability and a strong health system [67]. In the past decade, Ethiopia has rapidly expanded its health institutions, such as district hospitals, health centres and health posts, and deployed a large number of health professionals in all parts of the country. There is a need to emphasize the need for capacity building of health professionals, so that they can improve the diagnostics, including the use of molecular techniques, and good disease and vector surveillance [68].

The existence of health extension workers (HEWs) at grass root level in the Ethiopia health system is one of the opportunities to implement a mix of malaria prevention and control tools. In the Ethiopian health system, there is one community health post per *kebele* (the lowest government administrative unit having approximately 5000 population). The health post is staffed by two HEWs, and they are an important agent for malaria case management, can identify transmission foci, coordinate IRS and LLINs operation, perform surveillance and carry out information, education and communication to prevent malaria transmission. Moreover, HEWs can play a major role in mobilizing the community for larval control (environmental management) and in improving housing [69]. The other opportunity is the presence of a strong political commitment, national health policy, global partnership such as the RBM partnership, good global and international resource mobilization.

### Challenges

The main challenges for malaria elimination are the observed high prevalence of asymptomatic malaria infection [70], drug and insecticide resistance, climate change, political instability, a poor health system (with limited availability of diagnostic tools, frequent stock-outs of drugs, and varying and inadequate motivation of health workers), dependency on international donors, sustainable political commitment, behavioural challenges (example LLINs use), and weak surveillance, monitoring and evaluation. Moreover, the occurrence of *P. vivax* in Ethiopia makes elimination more difficult. This is because, *P. vivax* has a tissue dormant stage that can relapse after some time, and result in clinical infection that cannot be prevented by use of LLINs or IRS [71]. In the malaria guidelines of the Ethiopian Ministry of Health, the recommended drug of choice for radical cure of patients with *P. vivax* treated at health centres and hospitals in non-malarous endemic areas is primaquine [62]. However, there is a fear that primaquine can increase the risk of haemolysis in patient with glucose-6-phosphate dehydrogenase (G6PD). A recent nationwide survey concluded that the common G6PD*A-(G202A) or Mediterranean (C563T) variants of this gene deficiencies were not observed, and that the observed G6PD*A (A376G) mutation has little or no clinical significance [72, 73]. These findings supported the adoption in Ethiopia, primaquine for interrupting *P. falciparum* transmission and for radical cure of *P. vivax*. However, as the presence of other clinically important, less common variants cannot be ruled out [73], the implementation of radical cure should be accompanied by active haematological and adverse events monitoring.

## Conclusions

Currently available vector control tools such as LLINs and IRS can substantially reduce malaria transmission. Achieving and sustaining zero malaria transmission is unlikely without additional innovation, particularly in the presence of residual malaria transmission, insecticide resistance and asymptomatic malaria. Ivermectin administration, zooprophylaxis, odour-baited mosquito trapping systems, improving housing and larval control measures can be potential options for reducing malaria transmission.

## Abbreviations

ACT: artemisinin based combination therapy
DDT: dichloro-diphenyl-trichloroethane
GTS: global technical strategy for malaria
G6PD: glucose-6-phosphate dehydrogenase
HEW: health extension worker
IRS: indoor residual spraying
ITPs: insecticide-treated plastic sheeting
IVM: ivermectin
LLINs: long lasting insecticidal nets
MDA: mass drug administration
PBO: piperonyl butoxide
RBM: roll back malaria
Ro: basic reproduction number
WHO: World Health Organization
